# Global meta-analysis reveals agro-grassland productivity varies based on species diversity over time

**DOI:** 10.1371/journal.pone.0200274

**Published:** 2018-07-10

**Authors:** Amanda J. Ashworth, Heather D. Toler, Fred L. Allen, Robert M. Augé

**Affiliations:** 1 United States Department of Agriculture, Agricultural Research Service, Poultry Production and Product Safety Research Unit, Fayetteville, Arkansas, United States of America; 2 Department of Plant Sciences, University of Tennessee, Knoxville, Tennessee, United States of America; USDA Agricultural Research Service, UNITED STATES

## Abstract

Ecological research suggests increased diversity may improve ecosystem services, as well as yield stability; however, such theories are sometimes disproven by agronomic research, particularly at higher diversity levels. We conducted a meta-analysis on 2,753 studies in 48 articles published over the last 53 years to test: if biological N_2_ fixation (BNF) supplies adequate nitrogen (N) for plant growth relative to synthetic fertilizers; how crop physiological traits affect legume-grass symbiosis; and, how cultural practices affect BNF over a range of soils and climates overtime (in polycultures versus sole grasslands). Globally, net primary productivity (NPP; total aboveground production response of grass and legume in higher-diversity treatments) increased 44% via legume associations relative to sole grass controls (including both with and without N fertilizer). Several moderating variables affected NPP including: (i) *plant photosynthetic pathway* (mixtures of C_3_ grasses resulted in a 57% increase in NPP, whereas mixtures of C_4_ grasses resulted in a 31% increase; similarly cool-season legumes increased NPP 52% compared to a 27% increase for warm-season legumes relative to grasslands without diversity); (ii) *legume life cycle* [NPP response for perennial legume mixtures was 50% greater than sole grass controls, followed by a 28% increase for biennial, and a 0% increase for annual legumes)]; and, (iii) *species richness* (one leguminous species in a grassland agroecosystem resulted in 52% increase in NPP, whereas >2 legumes resulted in only 6% increases). *Temporal and spatial effect sizes* also influenced facilitation, considering facilitation was greatest (114% change) in Mediterranean climates followed by oceanic (84%), and tropical savanna (65%) environments; conversely, semiarid and subarctic systems had lowest *Rhizobium*-induced changes (5 and 0% change, respectively). Facilitation of grass production by legumes was also affected by soil texture. For example, a 122% NPP increase was observed in silt clay soils compared to 14% for silt loam soils. Niche complementarity effects were greatest prior to 1971 (61% change), compared to recent studies (2011–2016; -7% change), likely owing to reduced global sulfur deposition and increased ambient temperatures overtime. These historical trends suggest potential for legume intercrops to displace inorganic-N fertilizer and sustainably intensify global NPP. Results herein provide a framework for ecologists and agronomists to improve crop diversification systems, refine research goals, and heighten BNF capacities in agro-grasslands.

## Introduction

Worldwide biodiversity is in decline, especially in agricultural systems. Positive legume-grass associations have been reported in grassland ecosystems [1, 2, 3], as well as in semi-natural agricultural grasslands [4–6]; however, research on increased species diversity in C_4_ systems has reported little to no NPP benefit in the humid-Southeastern U.S. [7, 8]. These studies indicate that variations in sward species diversity, stand age, and photosynthetic pathway of both companion (legume intercrop) and main crops (grass species) may affect NPP, N_2_ fixation capabilities, and nutritive quality for animal fodder systems. Therefore, understanding cumulative linkages between legume-intercropping in grasslands, both spatially and temporally, will allow for better understanding of the ability of legumes to replace inorganic fertilizer N in agrograsslands.

Improved plant productivity from polycultures is usually explained by niche differentiation and facilitation [9, 10], as well as the increased probability of including a highly productive species in a mixture [11]. Niche differentiation suggests that intercropping is advantageous for stable yields during intense weather events because different species are able to occupy niches due to differing root and plant architecture, nutrient and water acquisition, and N_2_ fixation, resulting in improved NPP. Specifically, leguminous species are known to host BNF through a symbiotic relationship with *Rhizobia* (soil bacteria), which form nodules in roots, wherein dinitrogen from the atmosphere is converted into ammonium (NH_4_^+^), a plant-available N form [12]. The majority of N (up to 71%) is transferred via rhizodeposition (decomposition of nodules and root tissue and exudation of soluble N compounds by roots) [13].

Despite perceived benefits of legume intercropping, this is often not practiced in annual ruminant fodder production; thus high levels of N inputs are used to increase crop yields and soil mineral nutrition [13]. Nitrogen fertilizers are labile sources that can be applied at targeted growth stages and are major elemental sources of nutritive crop needs worldwide. However, manufacturing synthetic N fertilizers (via Haber-Bosch) is energy-intensive, as breaking trivalent bonds of N (N≡N) requires high pressure (100–200 atm), high temperature (400–500°C), and large amounts of energy (8000 kcal kg^-1^ N) for production of fertilizer [14]. Consequently, fertilizer N represents up to 50% of operational costs for crop production [15]. As such, this carbon-negative input has pricing linked to petroleum markets. Repeated applications of synthetic N to cropland can degrade surface- and groundwater [16, 17], considering up to 60% of N fertilizer applied is not used by plants and thus lost to soil and air [13]. This reality creates food security and sustainability challenges, particularly in the U.S.’s largest agricultural land base, as grasslands account for 46.8% of all agricultural lands and is the single largest land-use category [18].

Systematic, quantitative reviews, or “meta-analyses” are carried out to ascertain response variable impacts of large, multi-study datasets derived from literature. Such systematic reviews provide a global synthesis of research, and are a promising analytical technique for assessing cumulative effects spatially and temporally [19]. Meta-analyses, like Bayesian statistics, assumes non-normal distribution and take into account prior distribution for describing uncertainty; however, biases may exist in terms of publication and research bias [20, 21]. Nonetheless, the value of meta-analysis is that means within each study act as replicates, thus increasing the statistical power and allowing for aggregated results across a range of soils and climates, rather than over just a few study years and locations.

Therefore, in this study we used a meta-analysis approach to determine: i) if legume intercropping with grasses increased NPP relative to conventional systems (with and without inorganic-N); ii) symbiotic effects of diversity within a pasture agroecosystem; iii) forage quality impacts associated with legume integration; iv) how legume and main crop physiological traits [i.e., photosynthetic pathway, frequency of reproduction (annual vs. perennial)] affected legume-grass symbiosis; and, v) how cultural practices (i.e., establishment method, irrigation, and number of harvests within a season) affected legume facilitation (i.e. NPP) over a range of soils and climates over time.

## Materials and methods

### Data collection

On 11 November 2015 we conducted a two-tiered search on the Web of Science Core Collection, CAB International, MEDLINE, Biological Abstracts, FSTA (Food Science and Technology Abstracts) and Zoological Record databases, using the ISI Web of Science search tool. A search of these records using selected terms (i.e., legume intercrop, grass, mixture, forage, agriculture, AND yield), resulted in a total of 791 unique publications (S1 Fig). Thereafter, 743 were excluded because they did not meet inclusion criteria [i.e., means for both intercrop and inter-crop treatments were not presented; sole grass (control) yields were not reported; article was a duplicate; article did not contain primary data (no review or book); articles were not obtainable using interlibrary loan services, were refereed articles, or were conference proceedings, research reports, and bulletins]. Therefore, 48 unique articles met our screening criteria. Articles spanned 53 years (primary studies are provided in S3 Fig).

Treatment means of NPP (total aboveground production) and fodder quality parameters including in vitro dry matter digestibility (IVDMD), neutral detergent fiber (NDF), and acid detergent fiber (ADF) for either grass only (control) or grass and legume polycultures] and number of replications (sample sizes) were collected for each study. For publications reporting multiple grass monoculture controls, effect sizes were computed using the control that was treated most similarly to grass + legume treatments. Controls were represented by sole grass controls either receiving no N or an inorganic-N rate (N input was entered as a moderating variable). If replications were listed in a range, the smallest value was assumed. In studies not reporting replications we assumed *n = 1* (unless standard errors or least significant difference were reported, in which case *n = 2* was used). In intercropping experiments with >1 grass genus and no reported individual grass value (control), average yield and/or forage quality value for grass monocultures was used as a control. In some instances, where data were provided in graphical form, means were extracted using WebPlotDigitizer [22].

Given concerns that, within a paper, trials may not be independent, we down-weighted studies by a factor of m^^0.5^ (where m = number of trials in a paper; assuming 0.1 correlation among studies), allowing us to model studies as independent rather than dependent as often proposed [23]. This reduction in study weight resulted in a decrease in correlation between more distant time points in repeated measures produced by first order autocorrelation. Therefore, papers with more trials are likely to have lower correlation among those studies [24]. In addition, multiple treatment combinations (e.g. if available, both sole fertilized and unfertilized NPP was used to compare polyculture treatments) from a single article were treated as independent studies (e.g. paired observations) and were represented as individual units in our meta-analysis. Similarly, when a single control group was compared with multiple treatment groups (e.g. same control data used in calculating multiple RR), the non-parametric variance was adjusted by dividing the control group sample size by c^^0.5^, where c is the number of ES in which the same control group data are factored [e.g., in an experiment with one control and three treatments, each having 4 replicates, the control sample size (4) was divided by 3^^0.5^] [25]. Similarly, when one treatment group was compared to >1 grass-only control group, its sample size was partitioned into the ES across control groups. This was done to avoid overweighting trials by incorporating the same experimental units into more than one ES value. These methods are often employed in plant biology meta-analyses [26–28]. As such, we derived 2,753 studies from 48 articles. Each LS mean recorded was considered independent. Variance adjustments were carried out by Eq 1 (Eq 1) where n_trt and n_ctr are the sample sizes of treatment (trt) and control (ctr) groups, m is the total number of trials from the paper, c is the number of ES into which the same control and/or treatment sample size was incorporated, and t is the number of harvests for which data were reported for one year. If an article reported a single trial ES, the equation reduces to (n+n)/(n*n), simple non-parametric variance [25].

[(n_trt/c^0.5+n_ctr)/(n_trt/c^0.5*n_ctr)*(1+(t-1)*0.5))*(m/t)^0.5] (1)

### Effect size and moderator variables

A meta-analysis of NPP and forage quality parameters was conducted to ascertain global impacts of more diverse grassland mixtures and to quantify legume intercrops’ ability to replace synthetic nitrogen in those systems. Treatment ES was evaluated, which was calculated as the natural logarithm of the response ratio (ln*R*) of the intercrop to no-inter-crop means (Eq 2)

ES=lnR=lnY/YNCC (2)

where *Y* and *Y*_NCC_ are means of intercrop treatments and no-inter-crop controls [25]. These were used to calculate cumulative intercrop ES across studies relative to sole grass controls (with or without fertilizer) [24]. The use of response ratios (RR) in meta-analyses is common [29–31], as it gives a standardized expression of treatment-induced changes that have direct biological significance. Log transformations are required to balance positive and negative treatment effects across RR (thereby maintaining symmetry) [24]. For our analysis of the RR, ln values above 0 indicated intercropping induced benefits in the parameter of interest; values below 0 indicated an adverse effect from the intercrop; whereas values of 0 signifies a lack of an intercrop effect. Cover cropping (a crop planted in rotation to main crop for soil conservation) was not deemed equivalent to intercropping in this meta-analysis.

In addition to metrics associated with NPP (yield) and forage quality, we recorded information from each study on several moderators, or characteristics that may affect grass response to intercropping (Tables 1 and 2). Each moderator had at least two categories (levels) and data within each level were collected from at least 3 publications. These moderators were used as explanatory variables in the meta-analyses of overall summary effects. Moderators were chosen to determine if intercropping impacts are more pronounced under various externalities (e.g. more facilitative effects based on environmental conditions such as climate or soil texture). Possible temporal changes in ES were evaluated using harvest year after establishment as a moderator considering cumulative effects and N additions via rhizodeposition [32].

**Table 1:** Moderator analysis of intercropping influence on summary effects of grass yield.

Moderator	*Q*_*between*_^§^	n	*df*	*I*_*2*_ (%)	*p*_*hetero*_
	*Grass yield*				
Koppen climate classification	19.7	2728	7	0.0	0.006
Legume genus	23.5	2737	16	0.0	0.102
Grass genus	27.4	2737	16	0.0	0.037
Legume inoculant applied	1.0	2737	1	0.0	0.321
Relative nitrogen fertilization	10.8	2737	2	0.0	0.005
Legume life cycle	8.5	2142	2	0.0	0.014
Legume seasonality	4.2	2737	1	0.0	0.040
Number of legumes in mixture	9.8	2737	2	0.0	0.007
Number of grasses in mixture	9.7	2737	2	0.0	0.008
Soil texture	16.1	2676	7	0.0	0.024
Photosynthetic pathway of grass	9.9	2737	2	0.0	0.007
Seeding mechanism	4.4	2064	2	0.0	0.109
Irrigation	0.3	2737	1	0.0	0.597
Row spacing	4.3	1932	4	0.0	0.361
Control grass N fertilization	13.8	2695	4	0.0	0.008
Temporal treatment establishment	6.9	2667	5	0.0	0.226

^§^*Q*_*between*_, between-study variation (true heterogeneity); n, number of studies; *df*, degrees of freedom, levels within a moderator; *I*^*2*^, the ratio of true variation (heterogeneity) to total variation; *p*_*hetero*_ = p-value that all observed (total) variation is due to sampling error (within-study variation). Grass yield effect size is intercrop/no-inter-crop response ratio; analysis was conducted on log-transformed values (*lnR*) from each study. The levels of each moderator with their summary effect sizes, confidence intervals, intercrop-induced change as a percentage, number of studies and significance values are given in Figs 1–4; S4 and S5 Figs.

**Table 2:** Moderator analysis of intercropping influence on summary effects of forage quality (acid detergent fiber, neutral detergent fiber, crude protein, and in vitro dry matter digestibility).

Moderator	*Q*_*between*_^§^	n	*df*	*I*_*2*_ (%)	*p*_*hetero*_
		*In vitro dry matter digestibility*			
Legume genus	0.2	268	5	0.0	0.999
Grass genus	0.1	268	5	0.0	1.000
Legume inoculant applied	0.0	268	1	0.0	0.948
Legume seasonality	0.1	268	1	0.0	0.756
Photosynthetic pathway of grass	0.0	268	1	0.0	0.849
Irrigation	0.0	268	1	0.0	0.947
Temporal treatment establishment	0.1	244	2	0.0	0.964
		*Crude protein*			
Koppen climate classification	4.8	671	5	0.0	0.436
Legume genus	6.9	671	11	0.0	0.805
Grass genus	6.6	671	12	0.0	0.881
Legume inoculant applied	0.5	671	1	0.0	0.498
Relative nitrogen fertilization	0.2	671	1	0.0	0.640
Legume life cycle	1.2	671	2	0.0	0.552
Legume seasonality	2.2	671	1	0.0	0.143
Number of legumes in mixture	0.0	671	1	0.0	0.982
Number of grasses in mixture	0.2	670	2	0.0	0.990
Soil texture	19.7	650	5	0.0	0.001
Photosynthetic pathway of grass	2.4	605	1	0.0	0.477
Seeding mechanism	0.5	671	1	0.0	0.477
Irrigation	0.0	671	1	0.0	0.925
Row spacing	15.6	526	3	0.0	0.001
Control grass N fertilization	1.2	632	3	0.0	0.755
Temporal treatment establishment	22.5	655	5	0.0	<0.000
		*Neutral detergent fiber*			
Koppen climate classification	0.2	334	2	0.0	0.926
Legume genus	0.8	334	4	0.0	0.999
Grass genus	0.8	334	4	0.0	0.993
Legume inoculant applied	0.2	334	1	0.0	0.642
Relative nitrogen fertilization	0.3	334	1	0.0	0.613
Legume life cycle	0.4	334	2	0.0	0.821
Legume seasonality	0.0	334	1	0.0	0.865
Number of grasses in mixture	0.1	326	1	0.0	0.710
Soil texture	0.4	307	2	0.0	0.804
Photosynthetic pathway of grass	0.0	334	1	0.0	0.879
Irrigation	0.1	334	1	0.0	0.797
Row spacing	0.1	323	1	0.0	0.744
Control grass N fertilization	0.1	328	2	0.0	0.958
Temporal treatment establishment	0.6	324	2	0.0	0.728
		*Acid detergent fiber*			
Legume genus	0.1	144	2	0.0	0.9

^§^*Q*_*between*_, between-study variation (true heterogeneity); n, number of studies; *df*, degrees of freedom, levels within a moderator; *I*^*2*^, the ratio of true variation (heterogeneity) to total variation; *p*_*hetero*_ = p-value that all observed (total) variation is due to sampling error (within-study variation). Grass yield effect size is intercrop/no-inter-crop response ratio; analysis was conducted on log-transformed values (*lnR*) from each study. The levels of each moderator with their summary effect sizes, confidence intervals, intercrop-induced change as a percentage, number of studies and significance values are given in Fig 4; S4 and S5 Figs.

### Meta-analysis

Analyses reported herein followed the methodology of [24] and the criteria suggested previously [33]. A random-effects model was used in our meta-analysis, rather than a fixed-model (assumes a same value/true value for all studies). Summary effect (mean ES across studies) were estimated using Comprehensive Meta-Analysis (CMA) software (Version 3, Biostat, Englewood, NJ, USA; 2014). Individual studies within the meta-analyses were weighted using non-parametric variance [34]; Eq 3.

VlnRR=(nCC+nNCC)/(nCC*nNCC) (3)

where *V*ln*R* is the variance of the natural log of the *RR* and nCC and nNCC are samples sizes of the intercrop and no-inter-crop treatments [24, 32, 34]. Many publications do not report standard errors or standard deviations, or insufficient information is given in many instances to estimate variance via LSD or other mean separation values. It is not uncommon for measures of dispersion to not be reported in agricultural-focused publications, which makes calculating weighting based solely on sample size (non-parametric variance) a necessity [26, 34–36].

The *Q* statistic (or multiple significance testing across means; weighted squared deviations) was used to evaluate heterogeneity and was quantified using *I*^*2*^ (an index that estimates ratios of true variation: to total variation across ES) [29, 37]. *I*^*2*^ is defined as “(*Q*_*total*_—*df*) x 100/*Q*_*total*_, where *Q*_*total*_ is total variation; degrees of freedom (*df*) represents expected, within-study variation; and *Q*_*total*_—*df* is true heterogeneity, or between-study variation (*Q*_*between*_)” [25]. An *I*^*2*^ value of 0% = no true heterogeneity; >0 indicates true heterogeneity; and, larger values suggests variation due to true heterogeneity among studies. When *P* values for the *Q* test (*p*_*hetero*_) were less than 0.1, homogeneity assumptions were considered invalid [38].

### Potential data analysis bias

Publication bias applies to a body of research in refereed literature that is systematically unrepresentative of all completed studies [39]. Publication bias is more common in literature reviews, although this issue is raised more often with meta-analysis, likely because this method is intended to be quantitative and comprehensive [24]. The concern is that significant treatment differences are more likely to be published than non-significant findings. Direct evidence of publication bias is difficult to obtain, but it is important to check [32, 40]. Methods generally involve exploring the relationship between study ES and precision. The idea is that studies with smaller sample sizes or higher variance will have higher ES than larger studies with greater precision.

Begg and Mazumbar rank (Kendall) correlation [41] was used to evaluate publication bias. It was represented graphically with funnel plots of ES versus their standard errors [24]. A fail-safe method was used to asses if the summary effect may be attributing to bias. We employed the Orwin’s fail-safe N approach [24], considered an improvement on the original Rosenthal fail-safe N method [42]. The Duval and Tweedie iterative ‘trim and fill’ method was used to demonstrate how the summary ES would shift if apparent bias were to be removed [43]. Sensitivity analyses were also performed for the overall summary effects by removing one trial and re-running the meta-analysis for every trial, thereby illustrating how much each trial contributed to the summary effect, by noting how much summary effects changed. Possible temporal changes in the biomass summary effect were evaluated by examining how the summary effect has changed decade by decade.

## Results and discussion

### Potential data analysis bias

No evidence of publication bias was observed in our meta-analysis. Funnel plots for the influence of legume intercropping on NPP showed no pattern that would reflect bias toward not reporting small positive or negative effect sizes (ES; treatment/control). Large and small studies across the range of standard errors had the expected variability around summary ES. With the Begg and Mazumbar rank correlation test, NPP summary effect had an absolute Kendall value of 0.05, indicating no concern for publication bias (no tendency for ES to increase as study size decreases). The purpose of the fail-safe calculation is to estimate whether publication biases exist and if it can be ignored [44, 45]. The Orwin’s failsafe *N* was 1129; i.e. a very large number of missing studies would be needed to reduce the *P* value for biomass summary effect to >0.05 using a mean point (log) of 0.02 for missing trials. The stability of the overall summary effect was also assessed with sensitivity analyses i.e. the trial with the largest intercropping-induced change (i.e. ln*R* = -2.61) [46], was removed and changed the summary effect by 0.5% (from a 44.3% to a 44.8% increase in NPP).

**Fig 1:**
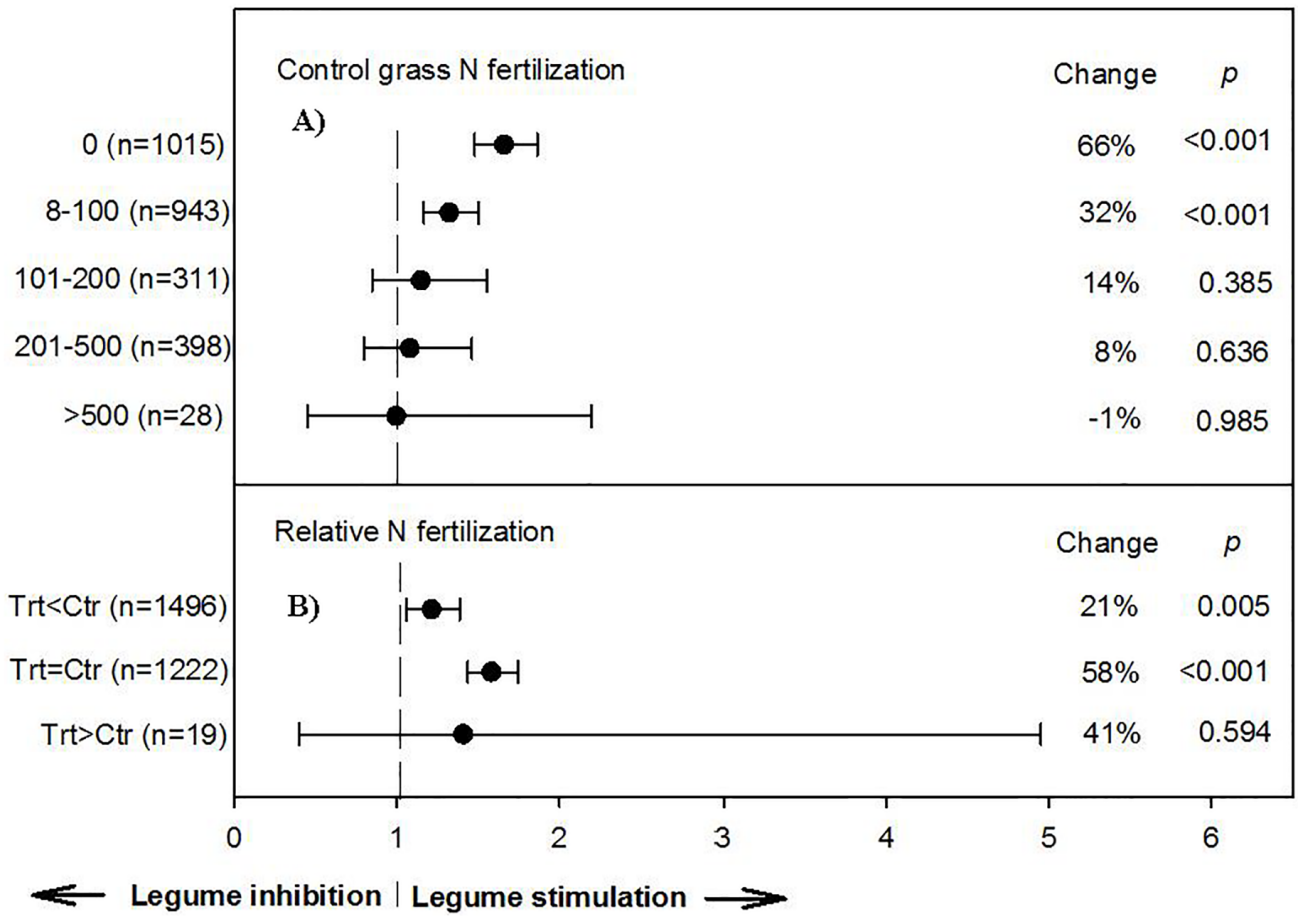
Weighted, overall summary effect sizes (response ratios) for NPP from legume-intercropping relative to sole grass fertilization rate in kg N ha^-1^ (a) and relative nitrogen fertilization (b). Negative values indicate inhibition from symbiosis, positive values indicate positive changes from the interaction. Change refers to raw percent affect in the effect size induced by legume-intercropping. Horizontal bars are 95% confidence intervals of the subgroup (moderator level) summary effect. *n* is number of studies contributing to the effect size. *P value* is the probability that the moderator level was statistically not different from zero.

**Fig 2:**
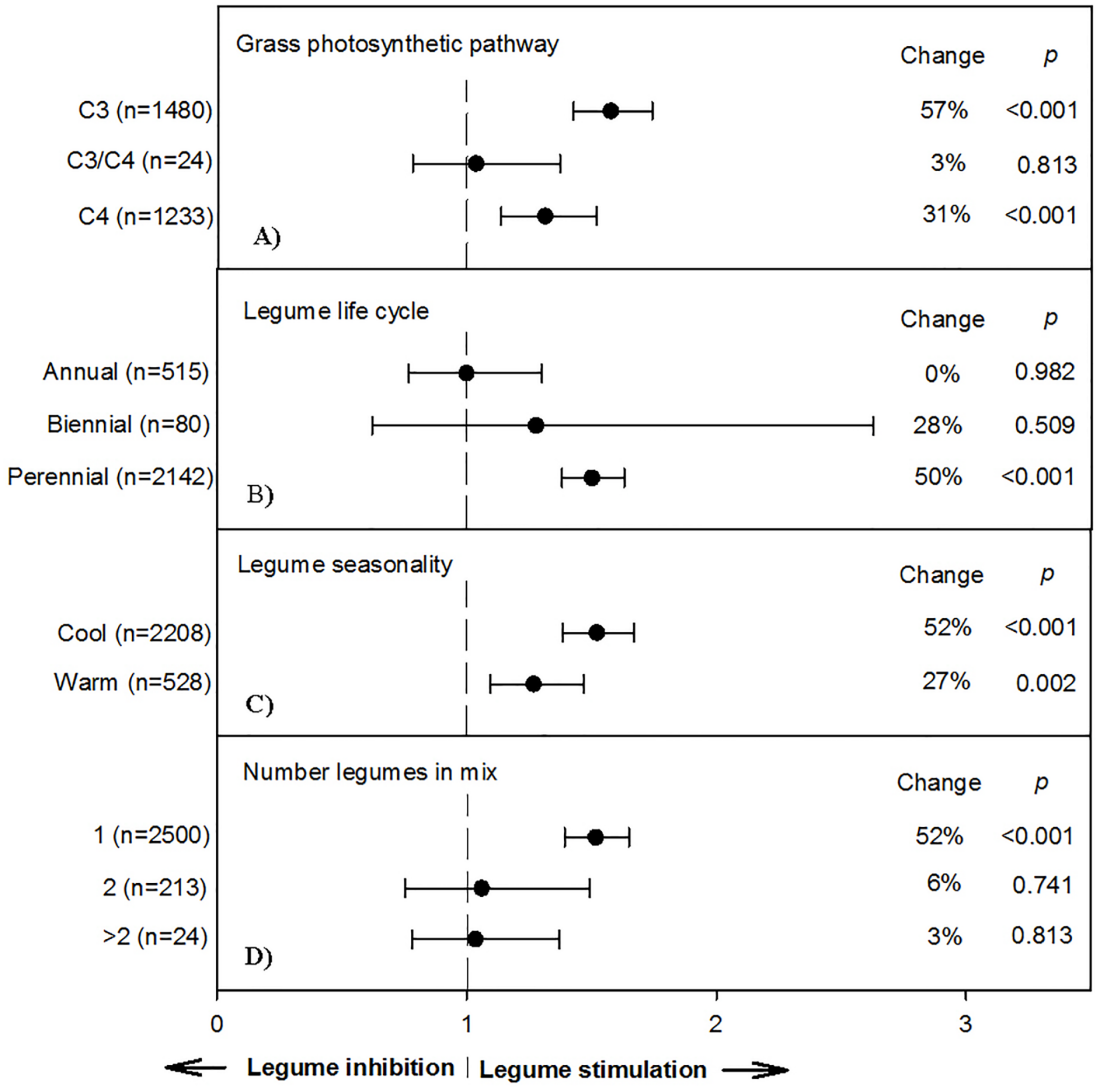
Weighted, overall summary effect sizes (response ratios) for primary productivity from legume-intercropping on grass photosynthetic pathway (a), legume life cycle (b), legume seasonality (c), and number of legumes in a mix (d). Negative values indicate inhibition from symbiosis, positive values indicate positive changes from the interaction. Change refers to raw percent affect in the effect size induced by legume-intercropping. Horizontal bars are 95% confidence intervals of the subgroup (moderator level) summary effect. *n* is number of studies contributing to the effect size. *P value* is the probability that the moderator level was statistically not different from zero.

**Fig 3:**
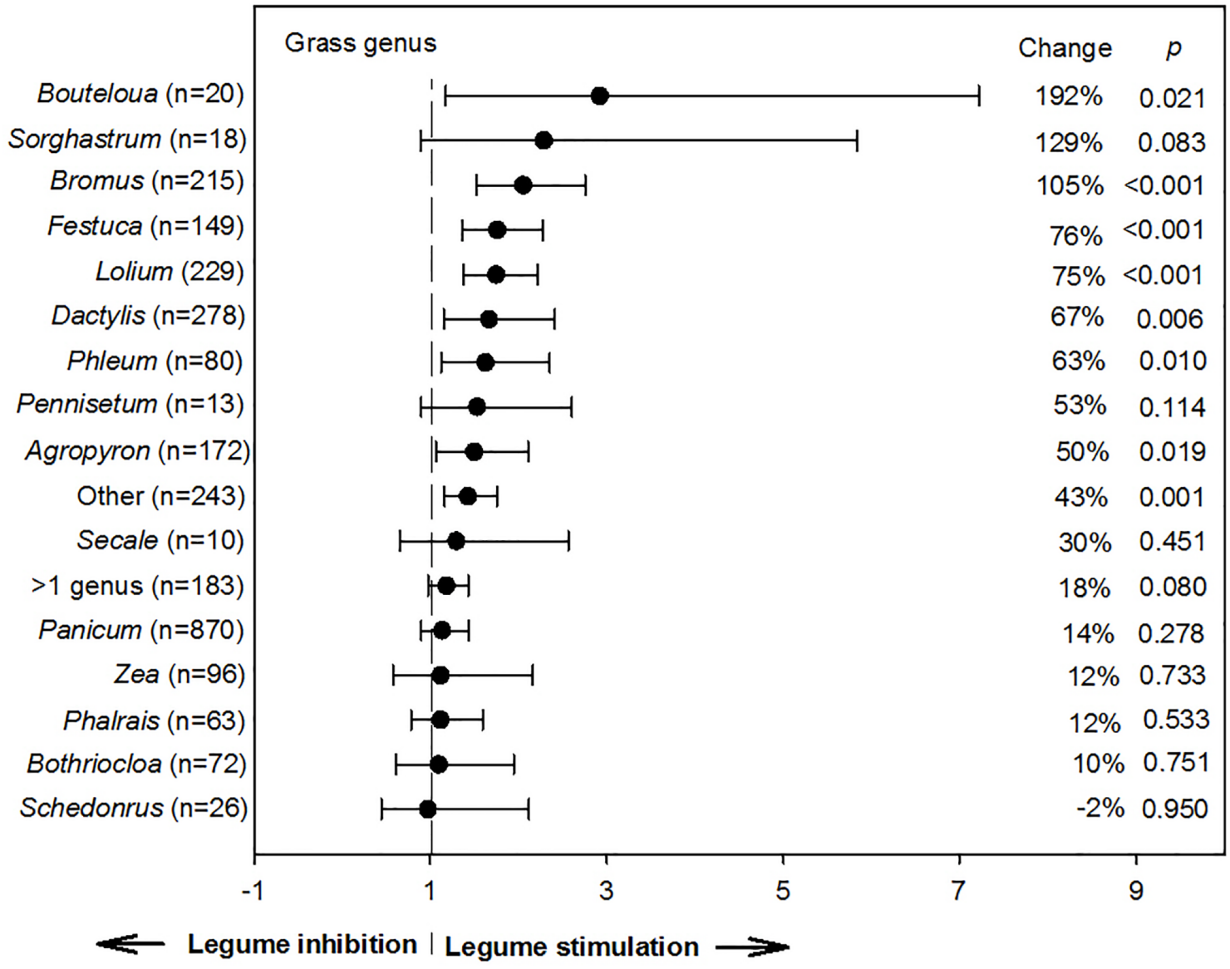
Weighted, overall summary effect sizes (response ratios) for primary productivity from legume-intercropping based on grass genera (negative values indicate inhibition from symbiosis, positive values indicate positive changes from the interaction). Change refers to raw percent affect in the effect size induced by legume-intercropping. Horizontal bars are 95% confidence intervals of the subgroup (moderator level) summary effect. *n* is number of studies contributing to the effect size. *P value* is the probability that the moderator level was statistically not different from zero.

**Fig 4:**
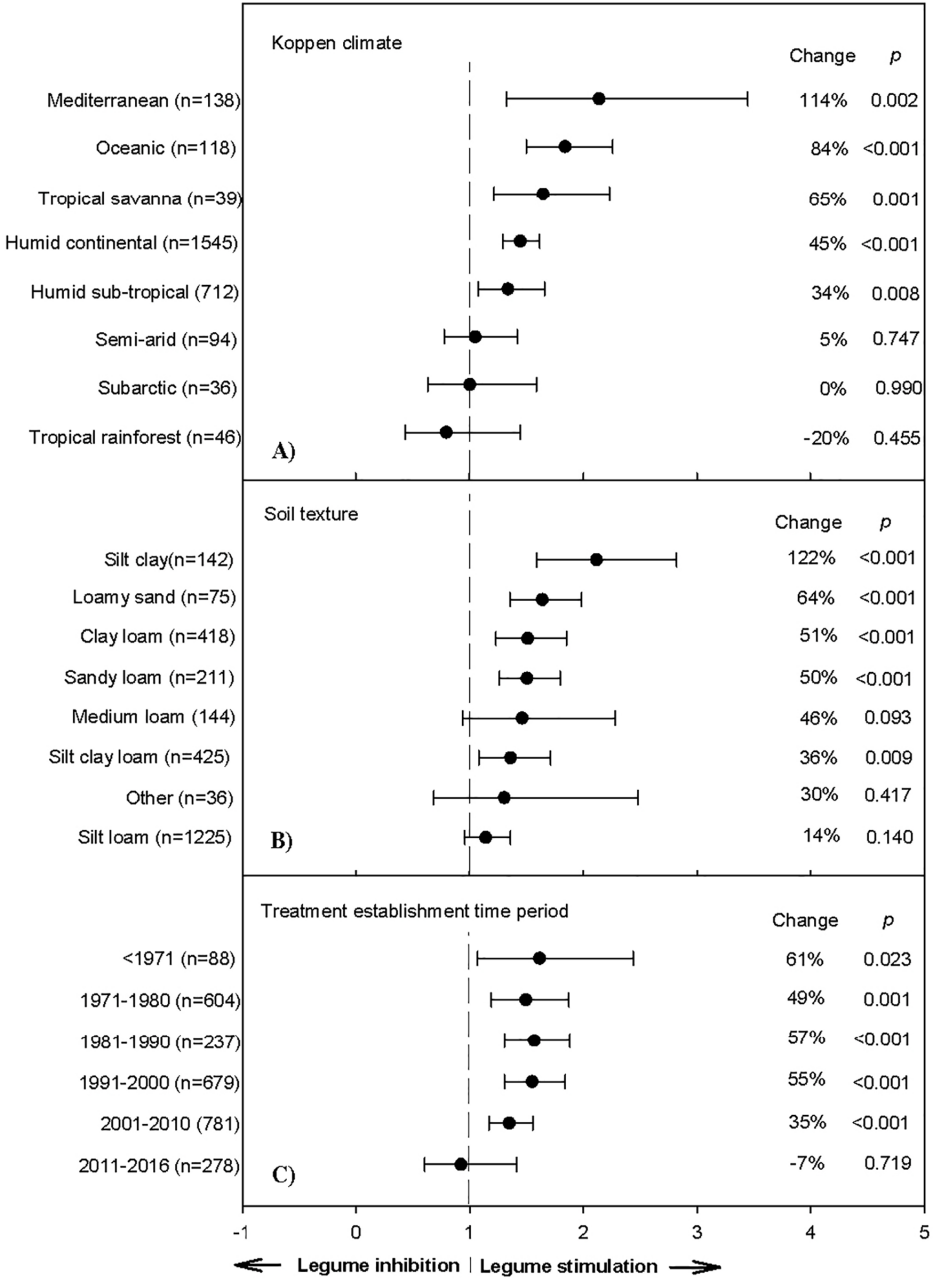
Weighted, overall summary effect sizes (response ratios) for primary productivity from legume-intercropping per Koppen climate classification (a), soil texture (b), and establishment period since 1971–2016 (c) (negative values indicate yield inhibition from symbiosis, positive values indicate positive changes from the interaction). Change refers to raw percent affect in the effect size induced by legume-intercropping. Horizontal bars are 95% confidence intervals of the subgroup (moderator level) summary effect. *n* is number of studies contributing to the effect size. *P value* is the probability that the moderator level was statistically not different from zero.

**Fig 5:**
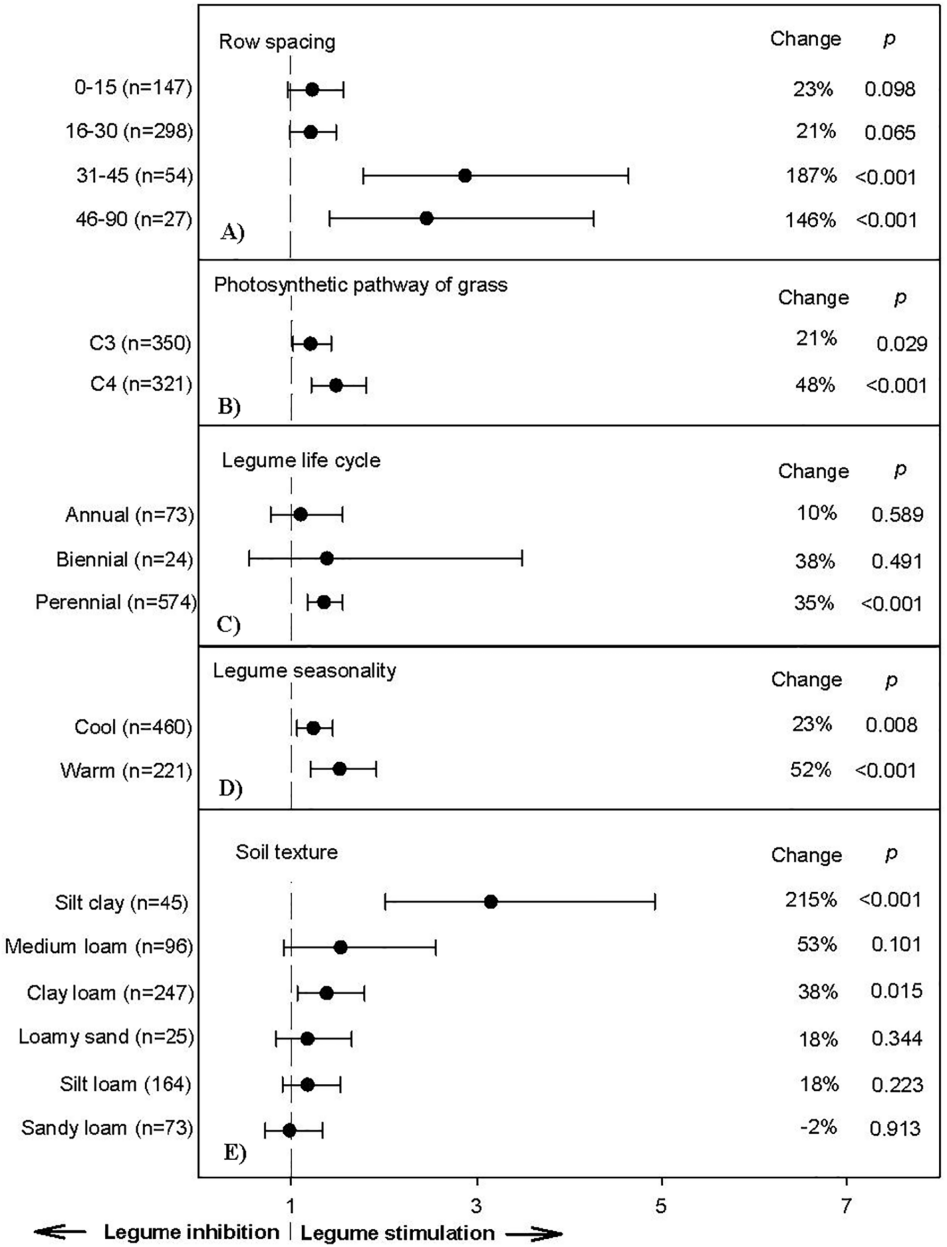
Weighted, overall summary effect sizes (response ratios) for crude protein from legume-intercropping based on system management including row spacing (a), photosynthetic pathway (b), legume life cycle (c), legume seasonality (d), and soil texture (e). Negative values indicate inhibition from symbiosis, positive values indicate positive changes from the interaction. Change refers to raw percent affect in the effect size induced by legume-intercropping. Horizontal bars are 95% confidence intervals of the subgroup (moderator level) summary effect. *n* is number of studies contributing to the effect size. *P value* is the probability that the moderator level was statistically not different from zero.

Analyses were conducted on natural logs and back-transformed to raw ratios. Summary of effect sizes—intercrop/no-intercrop RR—are depicted in forest plots (Figs 1–5). Percent change caused by intercropping, number of studies for each summary effect, and the statistical probabilities that summary effects are zero at *P*<0.05 are shown in forest plots. Statistical significance of summary effects is also denoted by confidence intervals (CI). If the CI does not cross the 1.0 vertical dotted line, they are significantly different than zero at *P*<0.05. It should be noted that in meta-analysis, unlike primary studies, the magnitude of the summary effect is regarded as of equal or greater importance than statistical differences [47]. Lack of statistical significance in meta-analysis is often due to insufficient numbers of studies and/or small sample sizes within experiments [48].

### NPP summary effects from diverse species mixtures

Several moderating variables were examined to determine moderator associations with leguminous intercropping with NPP and forage quality parameters. Heterogeneity statistics are included in Tables 1 and 2 for companion grass yield and forage quality, respectively. Over the 2,737 independent studies (also referred to as trials or paired observations in the meta-analysis literature; S1 and S2 Figs), RR suggests a positive NPP response globally from legume intercropping (44% NPP increase from sole grass controls; *P*<0.0001, CI: 0.0002–0.04).

More diverse plant communities, when compared to their sole grass counterparts, yielded 66% more total biomass when receiving no N-fertilizer (n = 1,105; Fig 1A). However, symbiotic effects were less pronounced when sole grass received 8–100 kg N ha^-1^, resulting in a 32% increase. Effects from intercropping continued to decrease with increased sole grass fertilization comparisons (101–200 and 201–500 kg N ha^-1^), resulting in 14 and 8% NPP increases, respectively (Fig 1A). Therefore, even at a higher monoculture (control) fertilization rate (100–500 kg N ha^-1^), N from grass-legume associations supplied comparative N via NDfA (nitrogen derived from the atmosphere) by nitrogenase enzymes; indicating use of this fertility source has the potential to maintain NPP while decreasing environmental degradation associated with inorganic-N fertilizer. Under an additional comparison, when controls received greater N rate than mixtures, intercrop systems still yielded 21% greater biomass; whereas when intercrop treatments and controls received the same amounts of N, mixtures produced 58% greater biomass (Fig 1B). Therefore, even assuming replacement of inorganic-N with BNF at a modest rate of one-third total consumption worldwide (5.6 million Mg NH_3_-N year^−1^), this would translate into a 15.1 million Mg NH_3_-N year^−1^ reduction of anthropogenic atmosphere emissions and $840M in savings for producers globally [49].

Grass photosynthetic pathway affected grassland mixture productivity and effectiveness of NDfA response (*P* = 0.007; Fig 2A). Grasses with C_3_ photosynthesis, when grown in polycultures with legumes, resulted in a 57% increase from grasslands with grasses alone, whereas C_4_ grasses resulted in only a 31% increase (Ln*R* = 0.27). Reduced facilitation of C_4_ grasses by legumes is consistent with higher N-use efficiency of C_4_ vs. C_3_ species, resulting in greater yields for monoculture grasslands, even under nitrogen-limiting systems [50]. Also, due to C_4_ species’ greater water-use efficiencies and overall greater photosynthetic rate per unit N (i.e. greater leaf expansion rates per unit N) [50], these species have greater compensatory competitive interactions [3], thereby resulting in reduced legume vigor and subsequent lesser fixation when grown in diverse mixtures. Similar to C_4_ grass response, warm-season legumes had reduced efficacy in mixtures (27% increase) compared to cool-season legume species (52%; Fig 2C), perhaps owing to more preferable senescence timing (optimal temporal rhizodeposition) and less competition from companion crops. Therefore, globally, mixtures of C_3_ grasses and cool-season legumes may result in the greatest facilitation among all grass-legume combinations.

Life cycle of the leguminous species in a mixture also affected grassland NPP (*P* = 0.014; CI: 0.32–0.48). Not surprising, perennial legumes, with assumedly better established and larger root systems (i.e. greater surface area for nodule root infection and greater yield stability during stochastic weather events) had higher total NPP responses. Specifically, polyculture yield response for perennial legumes was 50% greater than controls (all sole grass stands with and without fertilizer), followed by a 28% increase for biennial legumes, and a 0% increase for annual legumes (Fig 2B).

To test the overall effect of plant diversity in grassland agroecosystems, number of overall species in a mixture relative to monoculture grass systems was also tested (Fig 2D). Including only one legume resulted in the highest (*P*<0.05) percent increase in NPP (52%; n = 2,500), whereas two legumes in a mixture resulted in only 6% increases (Table 1). Such reductions could be due to inter and intra-specific competition due to dense grass plantings, thereby not allowing for niche differentiation or separation. This result was counter to that of [3], who reported that greater species-richness (up to 24 species total) leads to greater productivity. However, our meta-analysis indicates that in agrograsslands, having more than two species only increases productivity 3% relative to monoculture systems. Therefore, compensatory competitive interactions may have played a role in this relationship (i.e. relatively low productivity at higher diversity, considering dominating grasses likely have greater abundance in higher-diversity plantings due to compensation for poorly performing species).

A wide range of yield responses were observed for main crop grass genera (*P* = 0.037; Table 1), considering the summary effect was 2.9 and 2.3 x the magnitude of the control group for *Bouteloua* and *Sorghastrum* and only 0.98 for *Schedonorus* (192, 129, and -2% change, respectively; Fig 3). This suggests species with greater percent change may have greater: (i) nitrogen-use efficiencies (perhaps, in part due to the presence of associative, free-living diazatrophic colonization); (ii) scavenging potential of fibrous rooting systems; or, (iii) competitive growth which reduces fixation and lowers NPP responses from BNF. Overall, the effect size for legume genera did not impact (*P* = 0.102) NPP compared to sole grass controls (Table 2). However, trends suggest the greatest BNF efficacy resulted from *Desmodium*, *Medicago*, and *Lotus* intercrops (S4 Fig). Conversely, when *Vigna*, *Astragalus*, and *Onobrychis* were included in grassland mixtures they resulted in <10% increases from control yields, indicating a poor association from intercropping.

Overall, cultural practice summary effects for inoculation, irrigation, row spacing, and seeding mechanism varied for legume intercropping efficacy (S5A Fig). Surprisingly, inoculating did not improve global NPP (*P*>0.05; Table 1), considering a 51% of NPP was achieved with uninoculated in comparison to inoculated legumes. This result suggests inoculation failure or competitiveness of *in situ* free-living and associative microorganisms in the rhizosphere at time of planting [51]. In addition, across all environments and years, non-irrigated agro-grassland systems resulted in greater RR (45% change) compared to irrigated studies (3.4%; S5B Fig). Due to the high variance around summary effects of row spacing, non-detectable differences were observed (S5C Fig). However, the highest percent change (i.e. 70%) was observed for legume-grass row spacing of 0–15 cm, thereafter, efficacy declines occurred until 90-cm spacing. This result suggests closer row spacing (i.e. 0-15-cm) is optimal for heightened BNF transfer and rhizodeposition. Also, drilling legumes compared to broadcast seeding and transplanting tended to favor greater facilitation relative to grass only controls (51, 25, and 10%, respectively; S5D Fig).

#### Temporal and spatial influence on BNF efficacy

Legume symbioses fix approximately 70 million Mg of N per year worldwide, with about half this value occurring in temperate zones and the remainder taking place in tropical biomes [52]. This is likely owing to nitrogenase activity accelerating under ambient temperatures of 10 to 35°C, with varying temperature responses occurring among legume species [53]. Greater ambient temperatures also stimulate rhizodeposition due to death and decay of belowground tissues and exudation of soluble compounds. Therefore, not surprising, NPP responses to *Rhizobium* associations were most pronounced (114% change) in Mediterranean climates, followed by oceanic (84%), tropical savanna (65%), and humid continental (45%) environments based on the Koppen climate classification system (*P*<0.05; Fig 4A). Conversely, semiarid, as well as subarctic and tropical rainforest environments had lowest *Rhizobium*-induced changes (5, 0, and -20% change, respectively). The nitrogenase enzyme system has a very high activation energy of 2.18 eV; thereafter, potential rates of BNF decreases as temperature decreases to 22°C, and then falls rapidly under lower temperatures. Such kinetic differences likely contribute to the global pattern of BNF modelled by [54] and are confirmed in patterns reported herein (Fig 4A).

Free-living and dizatropic organism activity was also greatly affected by the seven represented soil textures (*P* = 0.024; Table 1), considering legume intercropping resulted in a 122% increase on silt clay soils over the control (Ln*R* = 0.75; Fig 4B). Silt clay soils have a high water-holding capacity and cation exchange capacity, resulting in high phosphorus retention, which is important considering the role phosphorus plays in legume nodulation. Despite being widely assumed that soil-water deficits severely depress *Rhizobium* activity [55], soils with >50% sand had a 50–64% greater NPP response. Notwithstanding low water-holding capacities, these soils were still hospitable for BNF. The lowest summary effect observed was for silt loam soils, as yield responses were 1.1x the magnitude of the control group (Ln*R* = 0.13; 14% change), indicating soils with greater propensities for greater N content have reduced BNF potentials due to grass monocultures being able to compete with legumes when soil nutrient levels are naturally higher, as they are more competitive under less stress-induced environments.

Testing whether a summary effect changes over time when trials that comprise the effect have been published over many years is recommended [20]. Changes in the summary effect could potentially result from publication bias, changes in methodology, or real biological changes. Investigating chronology (based on year of publication), suggests a decrease in N_2_ fixation efficacy in grass biomes over time (*P*<0.05; Table 1).

The niche complementarity effect [56] was greatest prior to 1971 (61% change), compared to recent studies (2011–2016; -7% change; Fig 4C). Facilitation has particularly declined since 2011, although this could be due in part to a smaller sample size (n = 278) and a large CI (Fig 4C). Response ratios have perhaps declined in part due to: i) 67% reductions in sulfur dioxide and subsequent deposition since the Clean Air Act of 1970 [57], considering that limitations of sulfur can restrict N_2_ fixation by affecting nodule development and function [58]; ii) greater N deposition coupled with greater ambient temperatures inhibiting legume photosynthesis and their competition in diverse mixtures; and, iii) a mean annual temperature increase of 0.9°C since 1970 [59], with subsequent increases in environmental stochasticity suppressing *Rhizobium* activity [55].

### Quality parameter summary effects from diverse species mixtures

#### Crude protein moderator impacts

Over 641 available trials, leguminous intercrops increased grass tissue-N by 32% (*P*<0.0001; CI: 0.14–0.40). This increase was not surprising considering N contributions from legumes to main crops may be as much as 50% of N via rhizodeposition [60]. Specifically, adding legumes as companion crops interacted with row spacing, soil texture, and time moderators to affect crude protein (CP) content in grass tissue (Fig 5), which has been found previously [61]. Surprisingly, greater grass-legume row distances had improved facilitation (e.g. 31–45 cm resulted in 187% greater CP levels compared to 16–30 cm distances only having a 21% increase). This suggests that a 31–45 cm legume-grass distance is optimum for maximum N-transfer and rhizodepostion via NDfA (Fig 5A), albeit this result is contrary to NPP results (S5C Fig).

Differences in polyculture species’ growth habit, management, photosynthetic pathway, and rate of legume maturity impacts the effects of the symbiosis on companion crop [7]. C_4_ grass polycultures had 48% greater CP when grown in mixtures compared to monocultures, whereas diverse C_3_ grass mixtures only increased CP by 21%. This was likely owing to C_4_ grasses having a higher photosynthetic rate per unit N_2_ compared to C_3_ species, because of the CO_2_ concentrating mechanism of C_4_ plants leading to CO_2_ saturation of rubisco [62]. Warm-season legume polycultures also had higher CP compared to their cool-season counterparts (52 vs. 23%), perhaps due to cool-season legumes not supplying peak N during main grass crop growth. Annual legume intercrops only increased CP 10% compared to a 38 and 35% for biennial and perennial species, respectively (Fig 5B). This suggests that greater rooting surface area may be linked to greater *Rhizobium* colonization. Soils with higher cation exchange capacity (i.e., silty clay, clay loam), compared to more drought prone textural classes (i.e., loamy sand and sandy loam) resulted in higher CP, suggesting greater nitrogenous activity and subsequent accumulation in these soils (Fig 5E).

#### Fodder digestibility impacts under greater diversity

Overall summary effects for IVDMD, NDF, and ADF were not affected (*P*>0.05) by intercropping despite an increase of 6% and decreases (lower values in this instance means greater digestibility and animal intake) of 9 and 6%, respectively. This was likely due to the overall low number of trials compared to other summary effects (268, 334, and 144, respectively). Consequently, moderators did not influence these forage quality parameters (Table 2). Despite summary effects not being affected, trends suggest that *Trifolium* intercrop spp. tended to reduce ADF and NDF, thereby resulting in less lignified mixtures with greater digestible fibers (S6 Fig). In addition, when *Medicago* spp. were included in diverse mixtures, higher IVDMD and improved NDF was observed compared to other legume species; suggesting greater digestibility and intake by herbivores, considering IVDMD increased 8% with a concurrent 10% decrease in NDF (S6 Fig).

## Conclusions

To continue to feed the Earth’s expected 9 billion population in 2050, it will be necessary to sustainably intensify agricultural production by 1.7 fold [63]. Legume intercropping may be one component of the management portfolio that reduces greenhouse gas emissions and chemical inputs, while maintaining NPP and fodder quality to the largest agricultural land base, agro-grasslands. Therefore, functionally diverse grass-legume mixtures may contribute to resource-efficient grassland systems and help mitigate climate change [61].

Our meta-analysis on 2,753 trials published over the last 53 years depicts strong evidence for the positive impact of plant diversification globally in pasture systems (44% increase in NPP via legume-*Rhizobium* associations), thus supporting the diversity-productivity hypothesis. However, a multitude of variables affect symbiosis efficacy, such as grass photosynthetic pathway, legume life cycle, cultural production practices, and species richness. Temporal and spatial effects also influenced BNF efficacy, considering BNF was greatest in less extreme environments based on the Koppen climate classification system.

These meta-analysis results demonstrate that grass-legume diversity promotes niche differentiation and facilitation, ultimately enhancing NPP and plant tissue-N for ruminating animals and improving agroecosystem sustainability. The framework provided herein on underling factors affecting the variability of BNF may help agroecologists develop functionally diverse systems to deliver ecosystem services while improving NPP.

## Supporting information

S1 Fig Checklist. (PDF)

S2 Fig PRISMA flow diagram for data inclusion in the meta-analysis. (JPG)

S3 Fig References of data used in the meta-analysis. (PDF)

S4 Fig Weighted, overall summary effect sizes (response ratios) for yield from legume-intercropping based on legume genera (negative values indicate inhibition from symbiosis, positive values indicate positive changes from the interaction). Change refers to raw percent affect in the effect size induced by legume-intercropping. Horizontal bars are 95% confidence intervals of the subgroup (moderator level) summary effect. *n* is number of studies contributing to the effect size. *P value* is the probability that the moderator level was statistically not different from zero.
(TIF)

S5 Fig Weighted, overall summary effect sizes (response ratios) for yield from legume-intercropping based on system management including legume inoculation (a), irrigation (b), row spacing (c), and seeding mechanism (d). Negative values indicate inhibition from symbiosis, positive values indicate positive changes from the interaction. Change refers to raw percent affect in the effect size induced by legume-intercropping. Horizontal bars are 95% confidence intervals of the subgroup (moderator level) summary effect. *n* is number of studies contributing to the effect size. *P value* is the probability that the moderator level was statistically not different from zero.
(TIF)

S6 Fig Weighted, overall summary effect sizes (response ratios) for in vitro dry matter digestibility, and natural and acid detergent fiber from legume-intercropping based on legume genera (negative values indicate inhibition from symbiosis, positive values indicate positive changes from the interaction). Change refers to raw percent affect in the effect size induced by legume-intercropping. Horizontal bars are 95% confidence intervals of the subgroup (moderator level) summary effect. *n* is number of studies contributing to the effect size. *P value* is the probability that the moderator level was statistically not different from zero.
(TIF)

## Abbreviations

ADF: acid detergent fiber
BNF: Biological N_2_ fixation
CI: confidence intervals
CMA: Comprehensive Meta-Analysis
CP: crude protein
ES: effect size
*lnR*: natural log of the response ratio
NDF: neutral detergent fiber
NDfA: nitrogen derived from the atmosphere
NPP: net primary productivity
RR: response ratio
