# Correction: Global meta-analysis reveals agro-grassland productivity varies based on species diversity over time

**DOI:** 10.1371/journal.pone.0233402

**Published:** 2020-05-14

**Authors:** Amanda J. Ashworth, Heather D. Toler, Fred L. Allen, Robert M. Augé

S1 Table is omitted from the list of Supporting Information. It can be viewed below. With this correction [1], all relevant data are now provided.

## Supporting information

S1 Table Intercropping meta moderator effects. (XLSX)
