# Supplementary figures and images for: Global meta-analysis reveals agro-grassland productivity varies based on species diversity over time
Source: PLoS One. 2018 Jul 10;13(7):e0200274. doi: 10.1371/journal.pone.0200274 (PMC6039048; doi:10.1371/journal.pone.0200274)

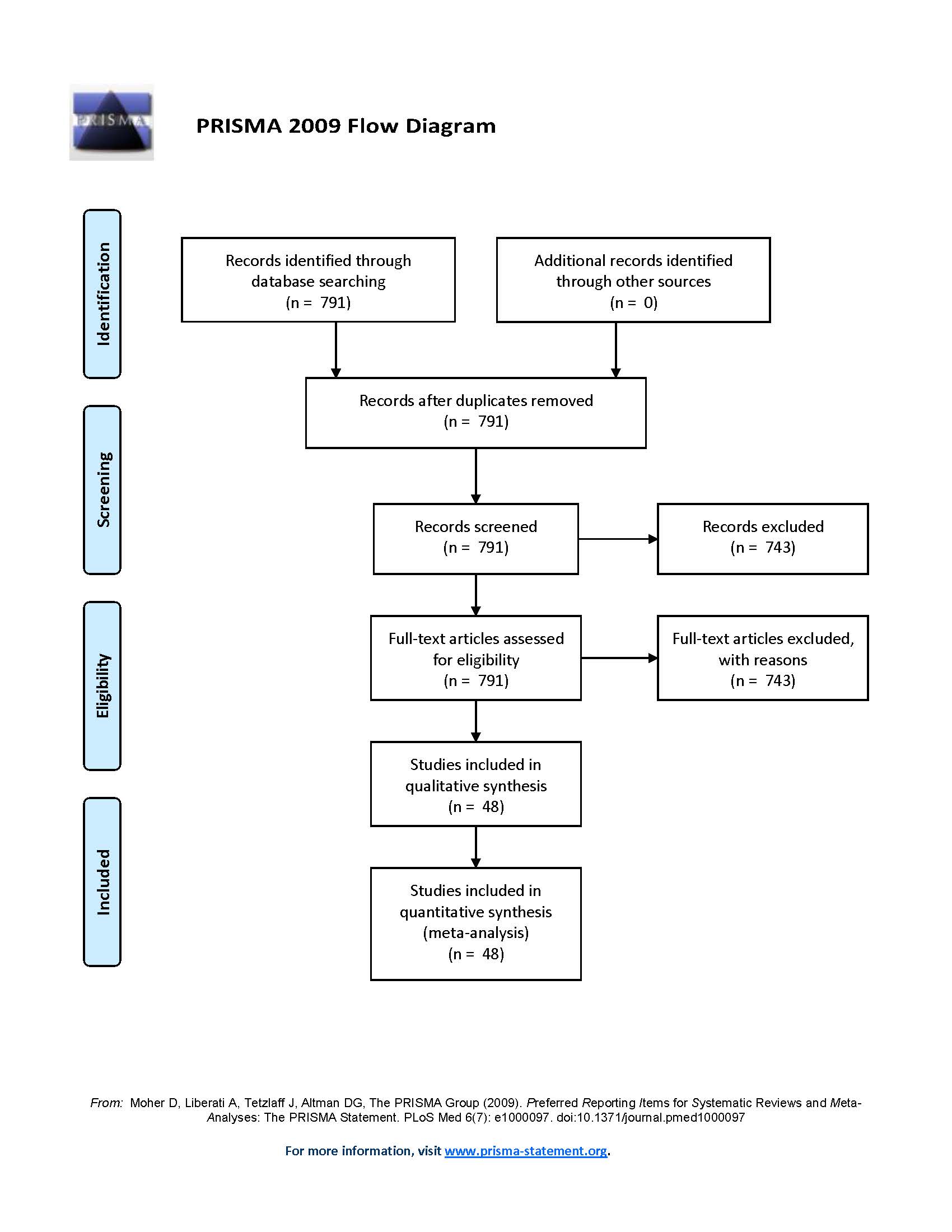

Supplement: S2 Fig — (JPG) [file pone.0200274.s002.jpg]

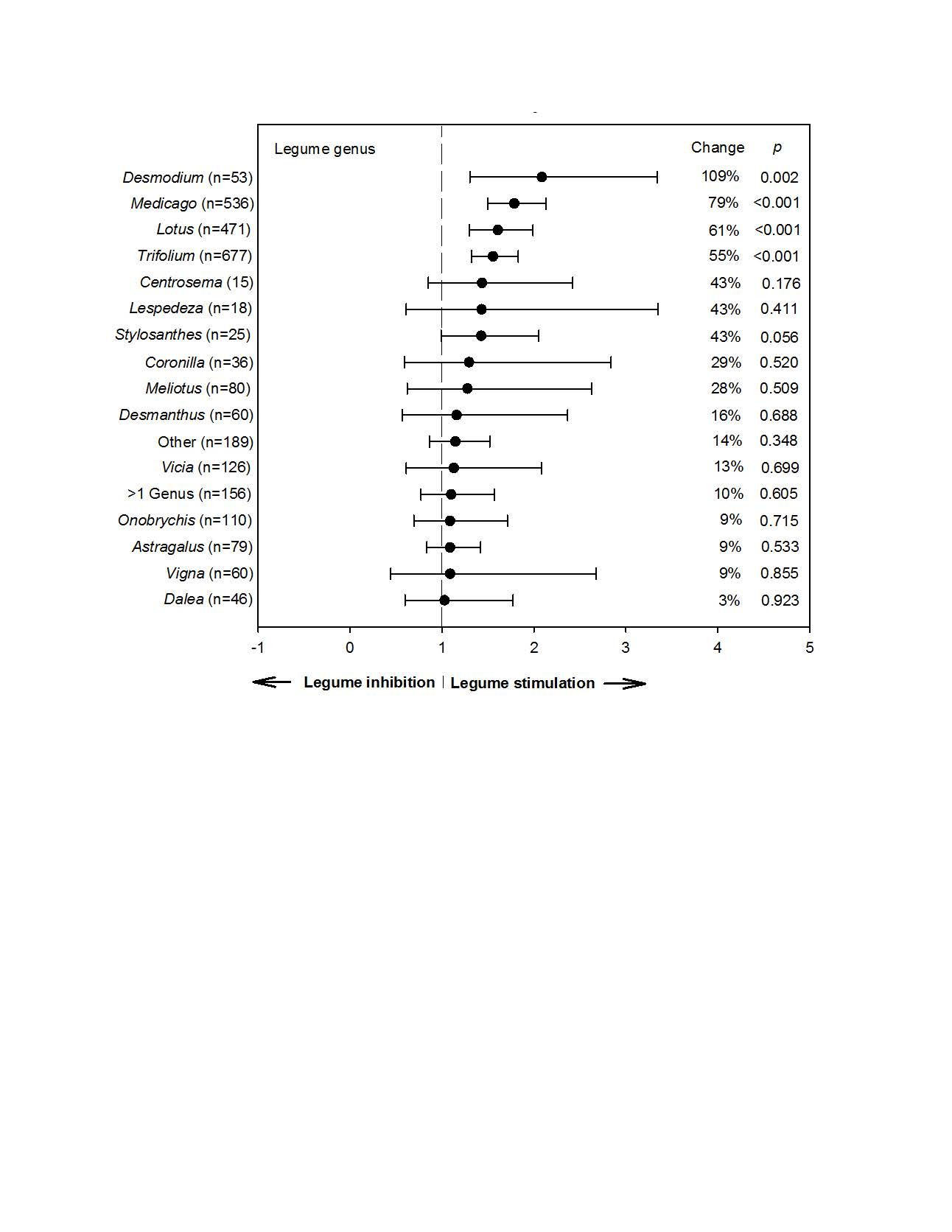

Supplement: S4 Fig — Change refers to raw percent affect in the effect size induced by legume-intercropping. Horizontal bars are 95% confidence intervals of the subgroup (moderator level) summary effect. n is number of studies contributing to the effect size. P value is the probability that the moderator level was statistically not different from zero. (TIF) [file pone.0200274.s004.tif]

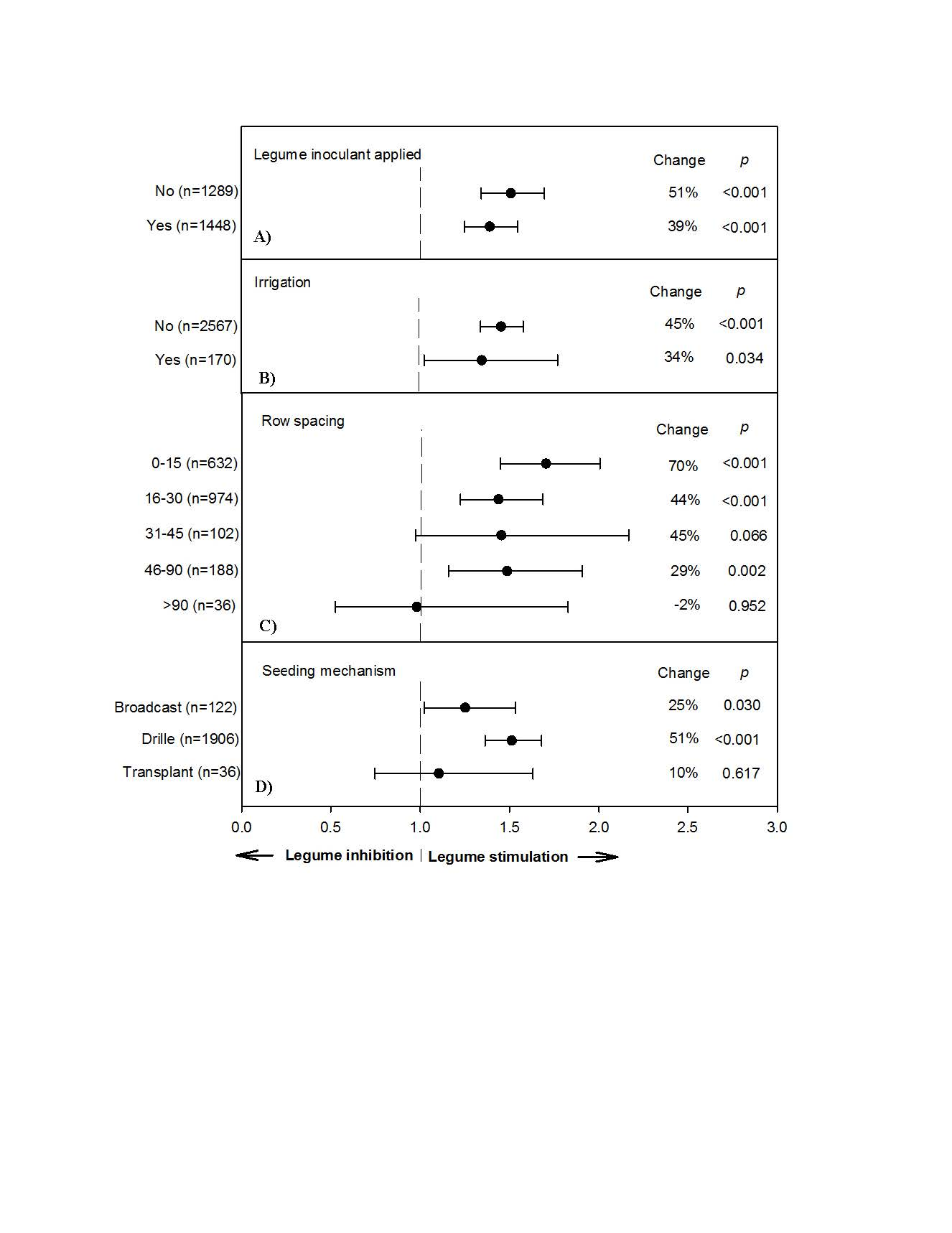

Supplement: S5 Fig — Negative values indicate inhibition from symbiosis, positive values indicate positive changes from the interaction. Change refers to raw percent affect in the effect size induced by legume-intercropping. Horizontal bars are 95% confidence intervals of the subgroup (moderator level) summary effect. n is number of studies contributing to the effect size. P value is the probability that the moderator level was statistically not different from zero. (TIF) [file pone.0200274.s005.tif]

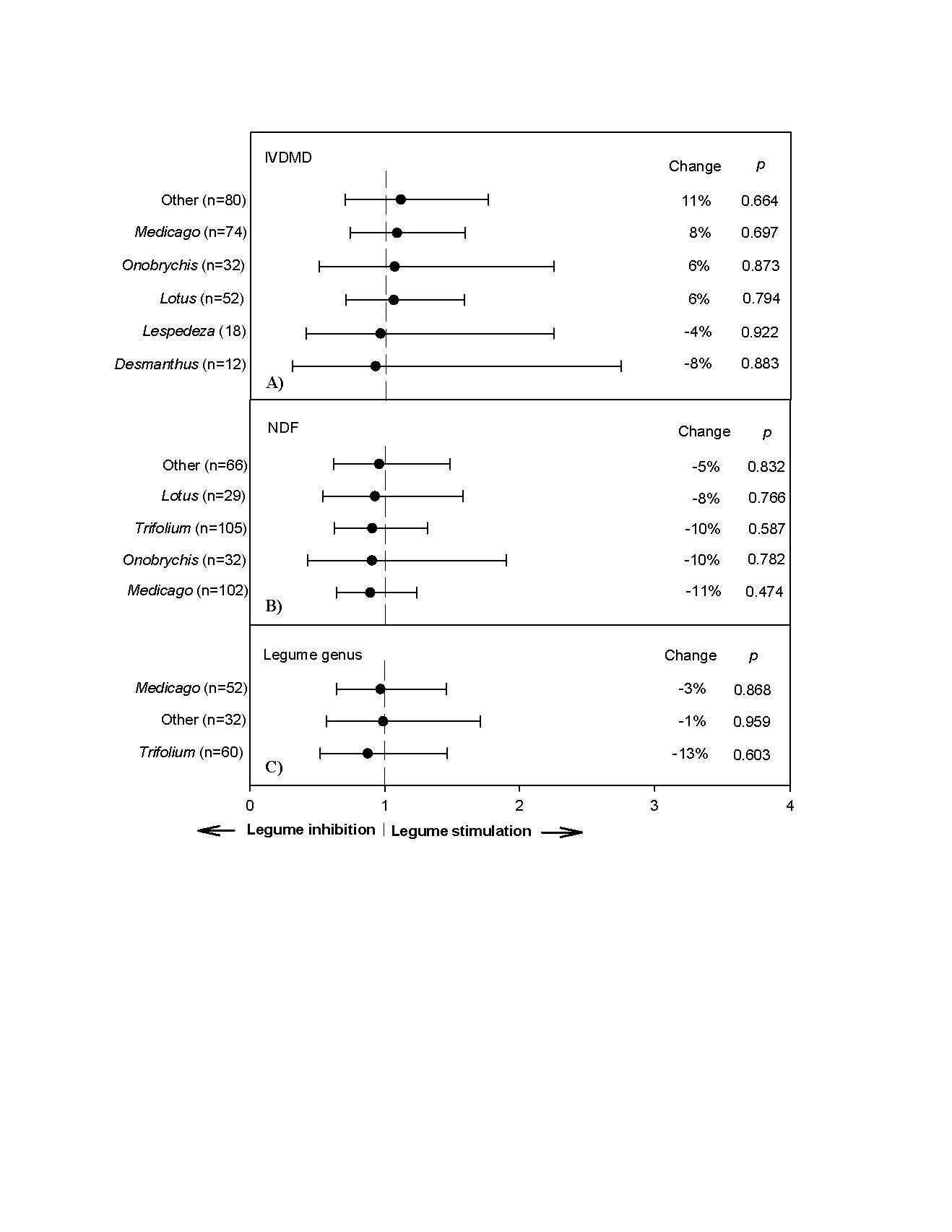

Supplement: S6 Fig — Change refers to raw percent affect in the effect size induced by legume-intercropping. Horizontal bars are 95% confidence intervals of the subgroup (moderator level) summary effect. n is number of studies contributing to the effect size. P value is the probability that the moderator level was statistically not different from zero. (TIF) [file pone.0200274.s006.tif]
