# Supplementary material for: Global meta-analysis reveals agro-grassland productivity varies based on species diversity over time
Source: PLoS One. 2018 Jul 10;13(7):e0200274. doi: 10.1371/journal.pone.0200274 (PMC6039048; doi:10.1371/journal.pone.0200274)
Supplement: S3 Fig — (PDF) [file pone.0200274.s003.pdf]

References of data used in the meta-analysis:

Adjei, M.B. 1995. Component forage yield and quality of grass-legume cropping systems in the Caribbean. Tropical Grasslands. 29: 142-149.

Ashworth, A.J., F. Allen, P. Keyser, D. Tyler, A. Saxton, and A. Taylor. 2015. Switchgrass yield and stand dynamics from legume intercropping based on seeding rate and harvest management. J. of Soil and Water Conservation. 40: 375-385.

Banski, T. 1990. Fertilization of grasslands with various ratios of legumes. Acta Agronomica Hungarica. 39: 65-71.

Barnett, F.L. and G.L. Posler. 1983. Performance of cool–season perennial grasses in pure stands and in mixtures with legumes. Agron. J. 75: 582-586. doi:10.2134/agronj1983.00021962007500040004x.

Biligetu, B., P.G. Jefferson, R. Muri and M.P. Schellenberg. 2014. Late summer forage yield, nutritive value, compatibility of warm- and cool-season grasses seeded with legumes in western Canada. Canadian Journal of Plant Sci. 94: 1139-1148. doi:10.4141/cjps2013-269.

Choudhary, V.K., A. Dixit, P. Suresh Kumar and B.S. Chauhan. 2014. Productivity, weed dynamics, nutrient mining, and monetary advantage of maize-legume intercropping in the Eastern Himalayan region of India. Plant Production Sci. 17: 342-352. doi:10.1626/pps.17.342.

Cui, S., V.G. Allen, C.P. Brown and D.B. Wester. 2013. Growth and nutritive value of three old world bluestems and three legumes in the semiarid Texas High Plains. Crop Sci. 53: 329-340. doi:10.2135/cropsci2012.02.0107.

Ezenwa, L., and M.E. Aken'ova. 1998. Performance of mixtures of selected grasses and adapted herbaceous legumes in south-west Nigeria. Tropical Grasslands. 32: 131-138.

Foster, A., C.L. Vera, S.S. Malhi and F.R. Clarke. 2014. Forage yield of simple and complex grass-legume mixtures under two management strategies. Canadian Journal of Plant Sci. 94: 41-50. doi:10.4141/cjps2013-095.

Frankow-Lindberg, B.E. and A.S. Dahlin. 2013. N-2 fixation, N transfer, and yield in grassland communities including a deep-rooted legume or non-legume species. Plant and Soil. 370: 567-581. doi:10.1007/s11104-013-1650-z.

George, J.R., K.M. Blanchet, R.M. Gettle, D.R. Buxton and K.J. Moore. 1995. Yield and botanical composition of legume-interseeded vs nitrogen-fertilized switchgrass. Agron. J. 87: 1147-1153.

Gierus, M., J. Kleen, R. Loges and F. Taube. 2012. Forage legume species determine the nutritional quality of binary mixtures with perennial ryegrass in the first production year.

Animal Feed Science and Tech. 172: 150-161. doi:http://dx.doi.org/10.1016/j.anifeedsci.2011.12.026.

Gokkus, A., A. Koc, Y. Serin, B. Comakli, M. Tan and F. Kantar. 1999. Hay yield and nitrogen harvest in smooth bromegrass mixtures with alfalfa and red clover in relation to nitrogen application. European Journal of Agron. 10: 145-151. doi:10.1016/s1161-0301(98)00061-6.

Hassen, A., L. Gizachew, N.F.G. Rethman and W.A. van Niekerk. 2007. Influence of undersowing perennial forages in maize on grain, fodder yield and soil properties in the sub-humid region of western Ethiopia. African Journal of Range & Forage Sci. 24: 35-41. doi:10.2989/102201107780178168.

Heichel, G.H. and K.I. Henjum. 1991. Dinitrogen fixation, nitrogen transfer, and productivity of forage legume-grass communities. Crop Sci. 31: 202-208. doi:10.2135/cropsci1991.0011183X003100010045x.

Hoveland, C.S. and M.D. Richardson. 1992. Nitrogen fertilization of tall fescue-birdsfoot trefoil mixtures. Agron. J. 84: 621-627. doi:10.2134/agronj1992.00021962008400040017x.

Jarchow, M.E. and M. Liebman. 2012. Tradeoffs in biomass and nutrient allocation in prairies and corn managed for bioenergy production. Crop Sci. 52: 1330-1342. doi:10.2135/cropsci2011.09.0481.

Jones, T.A., I.T. Carlson and D.R. Buxton. 1988. Reed canarygrass binary mixtures with alfalfa and birdsfoot trefoil in comparison to monocultures. Agron. J. 80: 49-55. doi:10.2134/agronj1988.00021962008000010011x.

Jungers, J.M., A.T. Clark, K. Betts, M.E. Mangan, C.C. Sheaffer and D.L. Wyse. 2015. Long-term biomass yield and species composition in native perennial bioenergy cropping systems. Agron. J. 107: 1627-1640. doi:10.2134/agronj15.0014.

Kunelius, H.T., G.H. Durr, K.B. McRae and S.A.E. Fillmore. 2006. Performance of timothy-based grass/legume mixtures in cold winter region. Journal of Agron. and Crop Sci. 192: 159-167. doi:10.1111/j.1439-037X.2006.00195.x.

Lauriault, L.M., S.J. Guldan and C.A. Martin. 2003. Irrigated tall fescue-legume communities in the southern Rocky Mountains: Years five to eight. Agro. J. 95: 1497-1503.

Lauriault, L.M., S.J. Guldan, C.A. Martin and D.M. VanLeeuwen. 2006. Performance of irrigated tall fescue-legume communities under two grazing frequencies in the southern Rocky Mountains, USA. Crop Sci. 46: 330-336. doi:10.2135/cropsci2005.0126.

Lauriault, L.M. and R.E. Kirksey. 2004. Yield and nutritive value of irrigated winter cereal forage grass-legume intercrops in the Southern High Plains, USA. Agron. J. 96: 352-358. doi:10.2134/agronj2003.1497.

Lindvall, E., A.-M. Gustavsson and C. Palmborg. 2012. Establishment of reed canary grass with perennial legumes or barley and different fertilization treatments: effects on yield, botanical composition and nitrogen fixation. Global Change Biology Bioenergy. 4: 661-670. doi:10.1111/j.1757-1707.2012.01178.x.

Lynch, D.H., R.P. Voroney and P.R. Warman. 2004. Nitrogen availability from composts for humid region perennial grass and legume-grass forage production. Journal of Environmental Quality. 33: 1509-1520.

Mallarino, A.P. and W.F. Wedin. 1990. Effect of species and proportion of legume on herbage yield and nitrogen concentration of legume-grass mixtures. Grass and Forage Sci. 45: 393-402. doi:10.1111/j.1365-2494.1990.tb01964.x.

McGinnies, W.J. and C.E. Townsend. 1983. Yield of three range grasses grown alone and in mixtures with legumes. J. of Range Management. 36: 399-401. doi:10.2307/3898498.

Mischkolz, J.M., M.P. Schellenberg and E.G. Lamb. 2013. Early productivity and crude protein content of establishing forage swards composed of combinations of native grass and legume species in mixed-grassland ecoregions. Canadian Journal of Plant Sci. 93: 445-454. doi:10.4141/cjps2012-261.

Ng, T.T. and T.H. Wong. 1976. Comparative productivity of two tropical grasses as influenced by fertilizer nitrogen and pasture legumes. Tropical Grasslands. 10: 179-185.

Pirhofer-Walzl, K., J. Eriksen, J. Rasmussen, H. Hogh-Jensen and K. Soegaard. 2013. Effect of four plant species on soil N^{15}-access and herbage yield in temporary agricultural grasslands. Plant and Soil. 371: 313-325. doi:10.1007/s11104-013-1694-0.

Posler, G.L., A.W. Lenssen and G.L. Fine. 1993. Forage yield, quality, compatibility, and persistence of warm-season grass—legume mixtures. Agron. J. 85: 554-560. doi:10.2134/agronj1993.00021962008500030007x.

Ram, S.N., S.S. Parihar. 2008. Growth, yield and quality of mixed pasture as influenced by potash levels. Indian J. Agric. Res. 42: 228-231.

Ranells, N.N. and M.G. Wagger. 1996. Nitrogen release from grass and legume cover crop monocultures and bicultures. Agron. J. 88: 777-882. doi:10.2134/agronj1996.00021962008800050015x.

Rasmussen, J., K. Soegaard, K. Pirhofer-Walzl and J. Eriksen. 2012. N_2-fixation and residual N effect of four legume species and four companion grass species. European J. of Agron. 36: 66-74. doi:10.1016/j.eja.2011.09.003.

Robert A. Zemenchik, K.A.A., and Melissa K. Schultz. 2001. Nitrogen Replacement Values of Kura Clover and Birdsfoot Trefoil in Mixtures with Cool-Season Grasses. Agron. J. 451-458.

Salama, H.S.A. 2015. Interactive effect of forage mixing rates and organic fertilizers on the yield and nutritive value of berseem clover (*Trifolium alexandrinum* L.) and annual ryegrass (*Lolium multiflorum* Lam.). Agricultural Sciences. 6: 415-425. doi:10.4236/as.2015.64041.

Sanderson, M.A., G. Brink, R. Stout and L. Ruth. 2013. Grass–Legume Proportions in Forage Seed Mixtures and Effects on Herbage Yield and Weed Abundance. Agron. J. 105: 1289. doi:10.2134/agronj2013.0131.

Schmidt, D.R. and G.H. Tenpas. 1965. Seasonal response of grasses fertilized with nitrogen compared to a legume-grass mixture. Agron. J. 57: 428-431.

Sebahattin Albayrak, M.T. 2012. Changes in the forage yield and quality of legume–grass mixtures throughout a vegetation period. Turkish Journal of Agriculture and Forestry. 37: 139-147. doi:10.3906/tar-1202-73.

Sengul, S. 2003. Performance of some forage grasses or legumes and their mixtures under dry land conditions. European J. of Agron. 19: 401-409. doi:10.1016/s1161-0301(02)00132-6.

Sollenberger, L.E., W.C. Templeton and R.R. Hill. 1984. Orchardgrass and perennial ryegrass with applied nitrogen and in mixtures with legumes. Grass and Forage Sci. 39: 255-262. doi:10.1111/j.1365-2494.1984.tb01690.x.

Simili da Silva, M., G.F. Tremblay, G. Bélanger, J. Lajeunesse, Y.A. Papadopoulos, S.A.E. Fillmore and C.C. Jobim. 2014. Forage energy to protein ratio of several legume–grass complex mixtures. Animal Feed Science and Technology. 188: 17-27. doi:10.1016/j.anifeedsci.2013.11.006.

Sleugh, B., K.J. Moore, J.R. George and E.C. Brummer. 2000. Binary legume-grass mixtures improve forage yield, quality, and seasonal distribution. Agron. J. 92: 24-29.

Springer, T.L., G.E. Aiken and R.W. McNew. 2001. Combining ability of binary mixtures of native, warm-season grasses and legumes. Crop Science. 41: 818-823.

Taylor, R.W. and D.W. Allinson. 1983. Legume Establishment in Grass Sods Using Minimum-Tillage Seeding Techniques without Herbicide Application: Forage Yield and Quality. Agron. J. 75: 167-172. doi:10.2134/agronj1983.00021962007500020003x.

Tessema, Z. and R.M.T. Baars. 2006. Chemical composition, dry matter production and yield dynamics of tropical grasses mixed with perennial forage legumes. Tropical Grasslands. 40: 150-156.

Thompson, D. 2013. Yield and nutritive value of irrigated tall fescue compared with orchardgrass: In monocultures or mixed with alfalfa. Canadian Journal of Plant Science. 93: 799-807. doi:10.4141/cjps2012-283.

Warwick, K., F. Allen, P. Keyser, A.J. Ashworth, D. Tyler, A. Saxton, and A. Taylor. 2016. Biomass and forage/biomass yields of switchgrass as affected by intercropped cool and warm-season legumes. J. of Soil and Water Conservation. 70: 374-385.

Whitney, A.S. and R.E. Green. 1969. Legume Contributions to Yields and Compositions of *Desmodium* Spp.-Pangolagrass Mixtures. Agron. J. 61: 741-746.
